# Global disparity in the supply of commercial weather and climate information services

**DOI:** 10.1126/sciadv.1602632

**Published:** 2017-05-24

**Authors:** Lucien Georgeson, Mark Maslin, Martyn Poessinouw

**Affiliations:** 1 Department of Geography, University College London, Pearson Building, Gower Street, London WC1E 6BT, UK.; 2 kMatrix Ltd., Greetham House, Greetham, Rutland LE15 7NF, UK.

**Keywords:** weather & climate services, weather & climate information services, weather services, climate services, climate data, weather data, climate risk, extreme weather risk, climate change, global framework for climate services

## Abstract

Information about weather and climate is vital for many areas of decision-making, particularly under conditions of increasing vulnerability and uncertainty related to climate change. We have quantified the global commercial supply of weather and climate information services. Although government data are sometimes freely available, the interpretation and analysis of those data, alongside additional data collection, are required to formulate responses to specific challenges in areas such as health, agriculture, and the built environment. Using transactional data, we analyzed annual spending by private and public organizations on commercial weather and climate information in more than 180 countries by industrial sector, region, per capita, and percentage of GDP (gross domestic product) and against the country’s climate and extreme weather risk. There are major imbalances regarding access to these essential services between different countries based on region and development status. There is also no relationship between the level of climate and weather risks that a country faces and the level of per capita spending on commercial weather and climate information in that country. At the international level, action is being taken to improve access to information services. With a better understanding of the flows of commercial weather and climate information, as explored in this study, it will be possible to tackle these regional and development-related disparities and thus to increase resilience to climate and weather risks.

## Teaser

Some weather and climate data are freely available, but major disparities exist in access to specialized information services.

## INTRODUCTION

Information about weather and climate is an essential service in modern societies, for example, for farmers that depend on seasonal forecasts to make decisions on crop choice, irrigation, and harvest dates (*1*) and for city governments and international companies that must design long-term climate change adaptation plans. A changing climate means that historical meteorological data and past climatic conditions are no longer sufficient to accurately analyze current and future hazard patterns (*2*, *3*). Climate change, alongside other contemporary drivers of global change, is already influencing world exposure to extreme weather events (*4*). For example, between 1970 and 2010, the human population exposed to annual flooding worldwide increased by 114%, significantly outpacing population growth over the same period (87%) (*5*). This is not solely a function of population growth and flood-related risks but also changes in where people are living; urbanization trends toward flood-prone cities and areas within cities may also increase exposure to flooding. It is argued that poverty reduction in a number of countries, particularly in Africa, has been “held back” by recent climate variability and extremes (*6*). Knowledge of short-term (weather) and long-term (climate) variations and uncertainties is essential for public and private sector organizations to make decisions and affects how billions of people live their lives.

Assessing risks from extreme weather events is vital not just for managing the risks from each event but also for strategic long-term decision-making. Data availability from many sources, including space-borne satellites, has increased markedly over the last decades, leading to a rapid expansion in weather and climate services. The data have often been provided free by governments and other publicly funded bodies, but interpretation and analysis of the data for specific needs is essential and is frequently provided by commercial organizations. When weather or climate data are combined with economic, demographic, or thematic data, they can be analyzed in a way that makes them relevant to the specific needs of public and private actors: from local authorities and smallholder farmers to government departments and multinational companies (*7*). We provide a unique analysis of the economic development of “weather and climate information services” (WCIS) over the last 5 years, showing which sectors and countries have the most access to this vital information.

Weather services are generally well known and understood. Forecasting has improved remarkably in recent decades. Five-day forecasts today are as accurate as 2-day forecasts 25 years ago and 5-day cyclone path forecasts have become the global standard; in the 1990s, even some 3-day forecasts were inconsistent (*8*). This is the basis of modern early warning systems for extreme weather events. Climate services have developed comparatively more recently, recognized by the launch of the Global Framework for Climate Services (GFCS) by the World Meteorological Organization (WMO) at the 2009 World Climate Conference (*9*). The GFCS is an international partnership to develop the global capacity for science-based climate services for decision-making and to address key challenges in capacity building, a lack of interaction between providers and users of data, the availability and quality of and access to data for decision-making, and the quality of climate services (*10*). The framework’s approach will be based on five pillars, including the novel “User Interface Platform,” which allows national weather services and other data providers (including the private sector) to work with those who use climate services to make them more relevant and useful (*11*). The other four pillars are as follows: Climate Services Information System; Observations and Monitoring; Research, Modeling and Prediction; and Capacity Development (*10*).

Our definition of WCIS is primarily focused on the flow and use of information, rather than the source of the data. There is a key difference between data and information (*12*); therefore, the huge increase in the quantity of data collected in the 21st century does not automatically lead to better information for decision-making. Moreover, although there is a difference between weather and climate information, they both exist on a continuum; many actors require both sets of information (*13*). We define weather information as generally more relevant in the short term or when immediate action is required, whereas climate information is more frequently required for longer-term planning and building climate resilience. WCIS data classification was based on five criteria: (i) allocation to “weather” or “climate” information, (ii) the platform of the service (such as “airborne services”), (iii) the industry type (such as legal and financial services), (iv) the subsector or market (such as reinsurance), and (v) the service type (such as “data management”). Here, the distinction between weather and climate information is based on the following definition. Weather is defined as shorter-term (days to a few years) information required for organization, operations, logistics, or profiling short-term risk. Climate information is defined as longer-term risk profiling with information required over decades.

Using the above classification, transactional data were collated on spending by private and public organizations on commercial WCIS. For example, a water company seeking information to inform future water course development may procure information services to assess how water courses and water storage may develop in the long term with changes in climate. The purchase of this project in the commercial WCIS market would be assigned to “climate services” for the relevant industry and subsector. Our methodology triangulates transactional and operational business data to estimate economic values in areas where government statistics are not available. Therefore, the result is a pragmatic assessment of the economic “footprint” of a commercial market for WCIS that only includes activities and data in the measurement of the sector where there is significant evidence to support it. The data triangulation methodology has been used previously to provide unique estimates in other emerging, hard-to-measure areas of the economy, such as climate change adaptation in cities (*14*) and carbon market intelligence (*15*). Data were measured for more than 220 countries and territories. A full description of the definition of WCIS and the data collection process is outlined in Materials and Methods.

## RESULTS

### Global results

**Fig. 1:**
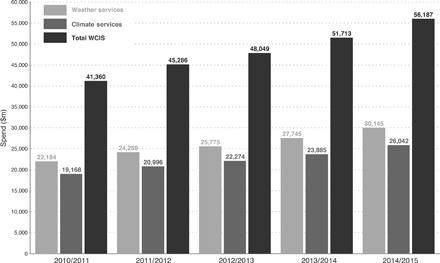
2010/2011 to 2014/2015 global spending on weather services, climate services, and WCIS (million dollars). Note that values are not adjusted for inflation and this should be considered when interpreting the growth in sales value between 2010/2011 and 2014/2015.

**Fig. 2:**
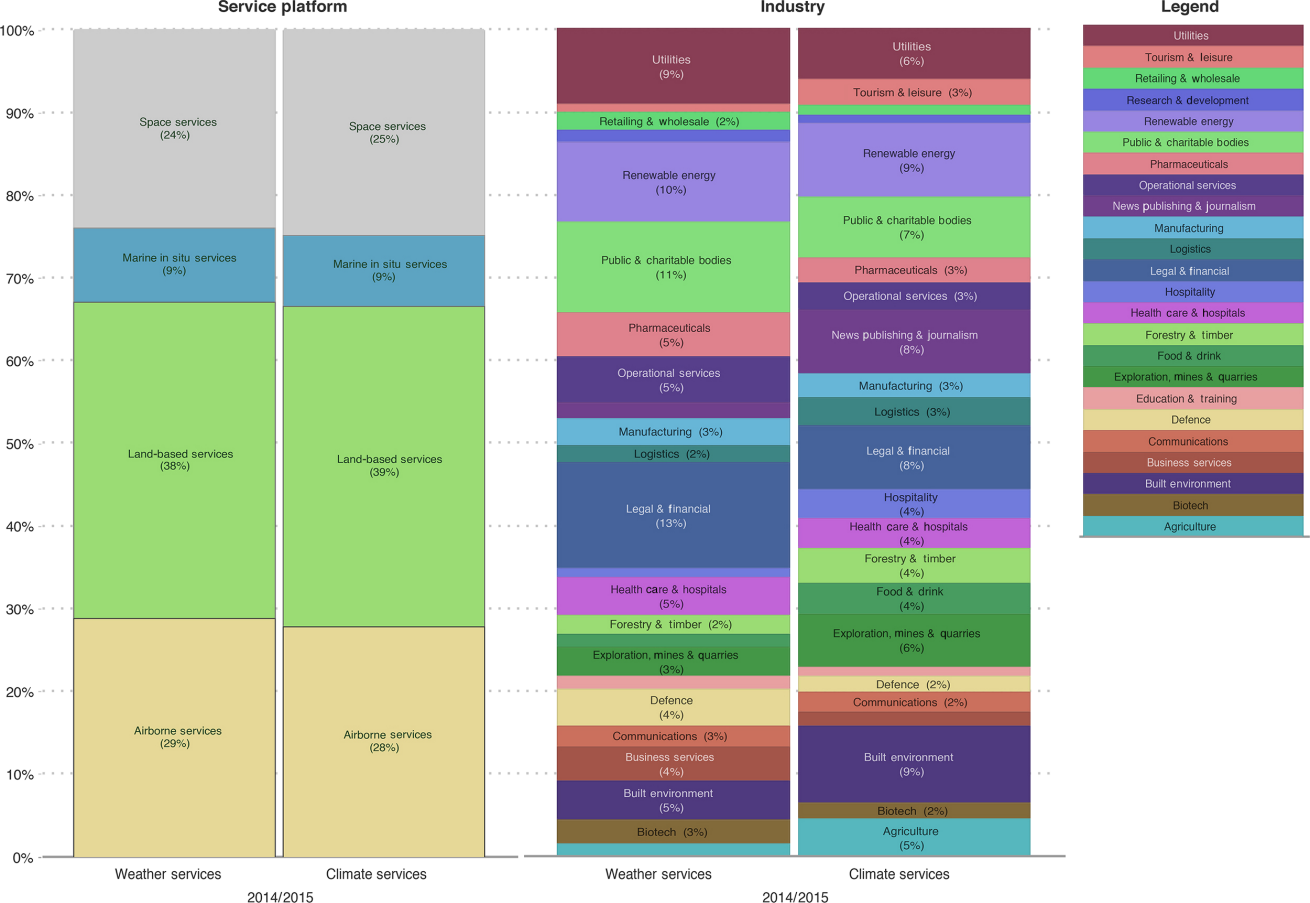
Sectoral breakdown of WCIS. By (**A**) platform of service (percent) and (**B**) industry (percent).

Total spend in 2014/2015 in WCIS reached more than $56 billion, with 54% spent in weather services and 46% in climate services (Fig. 1). This compares to an estimate of annual public funding for national meteorological services globally of $15 billion (*16*). Using the same transactional data methodology, we have estimated the global spending on adaptation to climate change in 2014/2015 at £223 billion ($357 billion) (*14*). This global measurement demonstrates that there is a significant and vibrant economic sector for commercial WCIS beyond freely available publicly funded weather and climate data. Figure 1 shows that WCIS are a small but significant sector of the global economy, which we estimate to be of the order of 0.08% of the global gross domestic product (GDP). Figure 2A indicates that satellite-based services (“space services” in Fig. 2A) are a significant part of the WCIS sector but that they represent only one-quarter of the total sector in terms of value. Satellite-based services provide a global perspective and new inputs into the analytical process (*17*), but marine, land-based, and airborne services are all also significant contributors to WCIS.

It is important to understand which organizations are purchasing WCIS. Figure 2 presents the sectoral breakdown for WCIS across 24 different industry sectors for both weather and climate services. There are significant variations in the share of weather versus climate information services across various industries, which may reflect the time scales over which most of the decisions have to be made in that industry. For example, built environment (weather, $1442 million; climate, $2414 million), agriculture (weather, $458.5 million; climate, $1183 million), forestry and timber (weather, $693 million; climate, $1088 million), exploration and extractives (weather, $1052 million; climate, $1675 million), and tourism (weather, $292.8 million; climate, $826 million) have a higher share of climate services. This may be because these sectors frequently need to make decisions with long-term consequences, such as those related to the location of economic activities and investment. In contrast, utilities (weather, $2755 million; climate, $1585 million) often need to respond to more immediate risks from extreme weather events and appear to have a higher need for weather services. Knowledge of weather impacts is likely to be very important to the pharmaceutical industry (weather, $1630 million; climate, $777 million) to understand what vaccines or treatments may be required where and in what quantities to respond to disease outbreaks. Legal and financial services (weather, $3861 million; climate, $2004 million) include the insurance market, which needs large amounts of weather-related information to assess risks to insured property, set rates, and assess claims. The share of both markets accounted for by public and charitable bodies is perhaps smaller because of the amount of publicly funded services; however, the fact that it represents a significant part of both areas of WCIS suggests that external verification or more expert analysis is also required by public bodies.

### Regional results

**Fig. 3:**
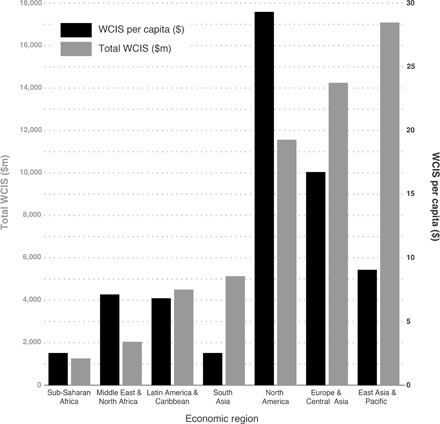
WCIS by region. Average per capita (dollars) spend in countries and total spend (million dollars) in WCIS grouped by region (192 countries).

The existence of a significant commercial WCIS market raises several questions about the distribution of these services. We found that there are significant differences in the scale of the commercial WCIS market between different regions and that spending on a per capita basis also varies significantly. Figure 3 presents a breakdown by region of where WCIS are being purchased. The World Bank’s regional classification was used to categorize the countries for which WCIS transactional data are available. The region that spends the most in total is East Asia and the Pacific ($16,500 million), whereas sub-Saharan Africa spends less than $1400 million. There appear to be very significant markets for WCIS in North America, Europe and Central Asia, and East Asia and the Pacific. The Latin America and Caribbean region has a lower total spend than East Asia and the Pacific and Middle East and North Africa. Figure 3 also presents the average per capita spend on WCIS for the countries of that region. The regional averages of per capita spending show some cause for concern about the disparities that exist in access to specialized weather and climate information across a number of regions, in relation to the number of people that may be vulnerable.

### Results by development status

**Fig. 4:**
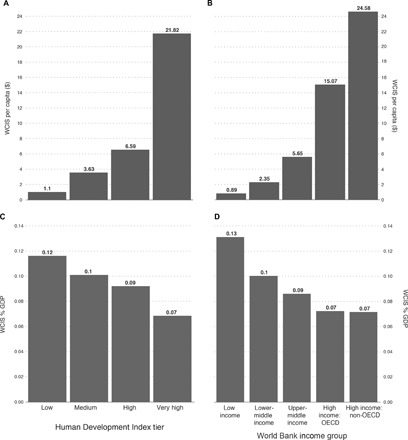
Relative spend in WCIS. WCIS average per capita spend (dollars) in countries grouped by developmental status: (**A**) UNDP HDI (179 countries) and (**B**) World Bank income group (185 countries). WCIS average spend as a percentage of GDP in countries grouped by developmental status: (**C**) UNDP HDI (183 countries) and (**D**) World Bank income group (190 countries).

This information does not give a clear indication of whether this is linked to development status however, because each of these world regions contains countries that face a variety of different socioeconomic situations. Figure 4A shows the mean of per capita spending for countries within the United Nations Development Programme’s (UNDP’s) Human Development Index (HDI). The HDI assesses countries across a number of socioeconomic indicators to give an assessment of the development status of each country. Figure 4B shows the mean of per capita spending for countries within the World Bank’s income classification, which is based on GDP per capita and is used to assess a country’s eligibility for World Bank funding and other statistical purposes. Figure 4 (A and B) suggests a strong relationship between an assessment of development status and per capita spending on WCIS. Notably, both present a marked difference between the highest strata of each category (“very high” on the HDI and high-income Organisation for Economic Co-operation and Development (OECD) countries on the World Bank’s income classification). On average, three times more ($21.36 per capita) is spent in the very high HDI countries compared to the “high” HDI countries ($6.59 per capita). In countries classified as “low income” by the World Bank, significantly less than $1 per capita is spent on average. However, Fig. 4 (C and D) shows that average WCIS spending as a percentage of GDP is significantly higher in less-developed than developed countries (0.13% of GDP compared with 0.07%). As actors in developing countries commit a higher percentage of their income to WCIS, despite the greater competing needs for their expenditure, this may be an indication of the importance that they give to WCIS.

### Weather and climate risks

**Fig. 5:**
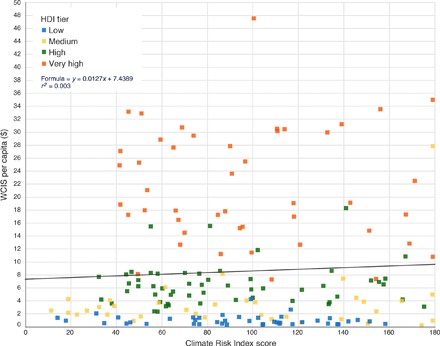
Climate Risk Index 1995–2014 score versus per capita spending on WCIS (dollars). Color-coded by HDI status (179 countries).

A more fundamental question is whether spending on WCIS is related to weather and climate risks that individual countries are exposed to, rather than their wealth. Figure 5 presents country per capita spending on WCIS plotted against each country’s score on the 2016 Global Climate Risk Index published by think tank and research institute, Germanwatch (*18*). The Climate Risk Index provides an analysis of exposure and vulnerability to climate-related risks (*18*). The scores from the Long-Term Climate Risk Index, which covers death toll, death toll per 100,000 inhabitants, total losses in million dollars, and losses per unit GDP in percent from 1995 to 2014, were used to provide a more comprehensive assessment of climate and extreme weather risk. A lower score on the Germanwatch Index indicates that a country was more affected by climate-related risks over the period of 1995–2014. The plots for each country have been color-coded by HDI status. There is no relationship between Climate Risk Index score and WCIS spend per capita. There appears to be a very slight inverse relationship with climate risk. However, the color coding shows a much stronger relationship between HDI status and WCIS per capita spending. This suggests that there needs to be an increased focus on capacity building and delivering tailored services for the most climate-vulnerable developing countries.

## DISCUSSION

It must be recognized that there are significant global efforts to develop publicly funded WCIS and to assist developing countries in WCIS capacity building. The European Copernicus Climate Change Service will be operational in 2018, delivering 33 “essential climate variables” on an open-access basis (*7*). The GFCS focuses on the most vulnerable countries, and the GFCS Taskforce initially made the recommendation that $75 million a year should be made available to implement the framework (*19*).

The High-level Taskforce for GFCS recognizes that there is a particular shortcoming in the current capabilities of WCIS in climate-vulnerable developing countries but also that WCIS need to be tailored to meet the needs of users (*19*). Decision-making in developing countries may be compromised if public and private organizations are reliant on less specialized free services, rather than specific, tailor-made commercial WCIS. The data presented in Results may suggest that decision-makers in less-developed countries may be more reliant on free data and services, which are not tailored to the specific problems that they face, because they have limited scope to pay for external suppliers of WCIS. Although there are a number of projects being carried out to help connect key users in developing countries with climate information and to assist them to interpret that information for decision-making (*2*), the extent to which this can counteract the potential lack of access to specialized services is not currently understood.

Funding is not the sole requirement for mobilizing greater deployment of WCIS globally, and the GFCS has a role in coordinating implementation of projects and leveraging the support of other actors. However, improvements in generating new funding for WCIS would appear to be important to overcome the disparity in access to the commercial WCIS market, especially in climate-vulnerable developing countries. In this area, the evidence is mixed; although the GFCS reported at the 2015 World Meteorological Congress that, in 16 of those countries, $700 million in total had been spent on climate services (*20*), it is not clear how much of this has been leveraged by the GFCS. As of June 2013, only $30.5 million had been pledged for GFCS funding (*21*). This suggests that WMO, the GFCS, and the wider international community need to find new strategies to mobilize additional funding aligned with GCFS priorities, rather than align it to investments that are already taking place.

Although it is difficult to ascertain an economic footprint for publicly funded provision of WCIS, one estimate suggested that total annual public funding of National Meteorological and Hydrological Services (NMHS) may exceed $15 billion (*16*). Case studies have consistently shown that the benefits of public investment in NMHS hugely outweigh the costs. The benefit-to-cost ratio of improvements in NMHS for disaster loss reduction ranges from 4 to 1 to 36 to 1 in developing countries (*22*). Benefits can vary between countries depending on the relative nature of climate risks, the people who have access to the services, and whether they have the capacity to respond, but it is clear from this study that there are significant additional social and economic benefits from WCIS. Services to deliver higher-quality, more specialized weather and climate information are vitally important for decision-making in many diverse areas, including health, disaster response, climate change adaptation, and many commercial sectors. The noncommercial services provided by the NMHS are only part of the overall picture.

A large and growing sector, analyzed here as WCIS, has emerged to provide more specialized and more specific data analysis services to a wide range of public and private actors across 226 countries and territories. The transactional data used in this study to measure spend in the commercial WCIS market suggest that a potential divide exists in the provision and availability of WCIS between those actors who can supplement the freely available services of certain NMHS by paying for additional information services in the commercial market for WCIS, and those actors that may lack the resources to do so. There must be some degree of concern as to whether the lower levels of spending in certain regions (Fig. 3) and in less-developed countries (Fig. 4), and the lower provision of information services that this suggests, affect the decisions that can be made. It is also of concern that there appears to be very little relationship between a country’s vulnerability to weather- and climate-related risks and the level of per capita spending on commercial WCIS (Fig. 5).

Although the transactional data in this study are not able to measure the “internalized” data and analysis of public and private actors, nor the large amounts invested in publicly funded, freely available data by some [but not all (*23*)] countries, the scale and consistency of the disparities in the commercial WCIS market demonstrate that a sizeable challenge exists in delivering more equitable, widely available, specialized information on weather and climate on a global scale. Initiatives such as the GFCS are intended to support by filling the gaps identified in this study but currently do not have the geographical scope or volume to address the pressing needs. Better design and delivery of WCIS have the potential to lead to better decision-making regarding weather and climate risks and challenges, and ultimately, this can help save lives and protect essential infrastructure. This study provides an improved understanding of the flows of weather and climate information beyond publicly funded data services; action is now required to tackle the apparent regional and development status–related disparities in the ability to access commercial WCIS.

## MATERIALS AND METHODS

The classification of WCIS was originally developed through a report for the UK Space Agency, the Met Office, and the Natural Environment Research Council (NERC) (and its industrial partners) in 2010/2011 (*13*). This research aimed to measure the global industry of WCIS, assess the platforms driving the industry, and assess which industries are driving the demand for WCIS. We improved the original classification system and extended the data cover from 2010 to 2015.

The main categorization was to distinguish between information services for weather and climate regarding the duration of time that the data or information services relate to: A short-term solution relies on weather information, whereas a long-term solution relies on climate data. However, weather and climate are, in essence, two points on the same continuum, and thus, previous categorizations that have solely referred to weather services or climate services risk providing an incomplete assessment.

In data collection, the following assumption was made on the distinction between weather and climate: Climate is the description of the long-term pattern of weather in a particular area. Some climate scientists define climate as the average weather for a particular region and time period, usually taken over 30 years; however, our definition was also informed by the way in which suppliers and users of information services define climate where possible. Frequently, when scientists analyze climate, averages of precipitation, temperature, humidity, sunshine, wind velocity, phenomena such as fog, frost, and hail storms, and other measures of the weather that occur over a long period in a particular place are analyzed. For example, after looking at rain gauge data, lake and reservoir levels, and satellite data, researchers can assess whether an area was drier than average during a summer. If it continued to be drier than normal over the course of many summers, then it would likely indicate a change in the climate. For measuring commercial WCIS, the purposes of these analyses can be varied; for example, a road stone organization procured information services related to the localized impacts on road surfaces in the future. This was allocated to climate because it requires a longer-term view for planning functions that need to assess how road surfaces may need to change as the climate changes.

### Defining WCIS

WCIS have been defined according to a broad definition of intent or purpose that takes into account all the proposed uses of WCIS put forward by the WMO (*19*). There is a difference between commercial and noncommercial WCIS. Governments have invested in infrastructure and research for short-term weather and long-term climate data, which governments often receive (and make available) free of charge. The WCIS sector, as a whole, therefore includes a significant amount of “free” data and information services that are internalized by public and private organizations. However, public weather and meteorological bodies also participate in the commercial market for WCIS. The data collection for WCIS for this study does not quantify the freely available or internalized publicly funded weather and climate data; these services do not have an economic footprint that would allow them to be measured. Public investment into WCIS is generally better understood (*22*), and therefore, this study confronts a major research gap in addressing the previously unmeasured and unreported commercial WCIS industry.

It is therefore a pragmatic statement on weather and climate services that collects and measures data only where sufficient evidence is available to support its inclusion in the broad definition of WCIS. The categories used in the data were generally derived from data sources in the industries that use those WCIS. The segmentation process and the headings used were mostly derived from the data sources themselves (the industries that are users of the products) and then grouped into a logical hierarchy based on the industry (such as, legal and financial services), subsector/markets (such as, reinsurance), service platform (such as, space services), and service type (such as, advisory services).

It is important to understand the ways in which data are being used to assess whether data collection is covering the full range of purposes involved in the procurement of WCIS. As part of the initial research phase, the uses of climate information were recorded and measured. However, these were not quantified in terms of the value of financial transactions because data sources did not show sufficient alignment on the definitions of these uses of information services. This process identified 26 different headings under which uses of WCIS could be aggregated, ranging from long-term corporate planning and major capital project planning to humanitarian relief planning and forward crop planning. This assessment of the uses of climate and weather data also assisted in the assurance process to underwrite the confidence that the data collection was capturing and understanding the main uses of weather and climate information.

The data sources related to purchasing (“buyer-side”) that were used to compile the research did not always share a clear distinction on whether they are seeking between a weather-related solution and a climate-related solution, whereas there was a greater level of alignment regarding purpose among “supply-side” data consulted. Therefore, a pragmatic approach was adopted on the basis of intent/purpose and time scale, that is, (i) whether the purchaser thinks the purchase is weather- or climate-related and, (ii) without “weather” or “climate” in the title, can the purchase or intent be identified as a short-term solution to a weather-sensitive issue or as a longer-term issue related to climate. For example, if an insurance company is looking to carry out a long-term risk-mapping exercise of weather and climate risks and spends approximately $6 million in a transaction for this service, this would be entirely allocated to climate services.

Each transaction was therefore assigned entirely to weather services or entirely to climate services, or the value was split between weather and climate based on the description of the purpose, and where necessary, allocation was based on the industry average uptake of those services (weather and climate) for that specific sector from buyer-side and seller-side data groupings for the relevant industry. For example, an events management procured information services regarding weather condition forecasts ahead of a major event in a transaction worth approximately $0.25 million. Because this requires a shorter-term view for planning an event, 93.7% of this transaction was allocated to weather based on the average industry uptake for that sector in relation to shorter-term decision-making. In this process, across all transactions, it is necessary to access both buyer-side and supply-side data groupings for the specific industry that is relevant to both the buyer and the seller. Without a fixed definition from the buyer-side data, a degree of interpretation is required to understand how definitions may vary between sectors, which also makes use of average industry uptakes based on the volume of data available. For example, corporate governance is one area of WCIS use that requires both long-term (climate) and short-term (weather) risk profiling; for an information service transaction that informed long-term locational conditions reporting, where buyer-side data were not conclusive, this transaction was allocated, with 73% to climate and 23% to weather, on the basis of industry average uptake for these services for this corporate’s specific sector.

Data were also sorted by platform, type of service, industry, and submarkets. The transactional data that comply with the processes identified were then categorized in a number of levels for accurate data processing and reporting: by four data platforms, eight types of service, 24 industrial sectors, and 114 market subsectors. Details of the categorization and examples from the data taxonomy for two sectors can be found in the Supplementary Materials. An example of the specific categories identified in this mapping process into which transactions are allocated would be climate advisory services from space-based services to the insurance sector. Examples of the types of transactions included in WCIS measurement in this study can also be found in the Supplementary Materials.

### Data collection and analysis

The methodology used for data acquisition and analysis was based on a system originally developed at Harvard University for triangulating transactional and operational business data to estimate economic values in areas where government statistics and standard industry classifications are not available (*24*). It has been used in a wide variety of public and private applications since then. A number of applications are publicly available, including geoservices (services reliant on geospatial data and images) (*25*), low carbon and environmental goods and services (*26*–*28*), the global water industry (*29*), and cybersecurity (*30*). The data triangulation method has also been used in other sectors (law and order, digital media and creative industries, commercial applications of marine environmental science, and agritech), but these reports are currently unpublished.

The new WCIS taxonomy was populated from the bottom up, searching for evidence for the ideal definition and including only elements where the evidence is available. kMatrix has, over the past 20 years, compiled more than 27,000 independent databases and sources to cover most global financial transactions. Each database or source is coded so that sector- and region-specific questions can be addressed. For this study, a subset of approximately 1000 relevant data sources was selected. The large number of data sources is essential because each transaction needs to be triangulated both with multiple sources and different types of measurements (sales, insurance value, and so on) to ensure its accuracy. The information on activity types and uses of WCIS provided the initial search terms; applying these search terms at the sectorial level of end users of WCIS identified further sector-specific terminology for identifying data sources and transactions. The process of searching and filtering is therefore iterative and incorporates sector-specific values to ensure that it is comprehensive.

The data triangulation methodology has a number of the characteristics of “big data” approaches (*31*): higher volume, higher velocity, and high variety. It uses a much higher number of sources than other approaches, delivers data for analysis more quickly, and needs to cope with data from a variety of sources in a number of different types.

For each transaction listed in the WCIS data, a minimum of seven separate sources must independently record the transaction for it to be confirmed and included in our database. At the country level, the average number of sources for each transaction is 131, ranging from 24 (Paraguay) to 224 (UK). These databases have been tracked and verified over a number of years. Using multiple sources of data and multiple types of data makes it possible to arrive at accurate estimates of transactional value that are not possible using a single source. The triangulation of data from multiple sources counteracts biases inherent in certain sources of data; this is also minimized because all sources are tracked and managed for accuracy and reliability over time. Given the range of industries covered, a wide range of data sources is required from a wide range of government, private, industrial (from major national and international industry associations to federations and trade bodies for specialized sectors and manufacturers), academic, financial, and other sources.

For WCIS, data are produced to an average confidence level of 82.5%; at the country level, average confidence levels for transactions range from 68 to 97%. Confidence levels are a function of the range of source values assembled for each data point. Each final data point is the mean of the final range of values (after outliers are removed). The confidence level is the difference between the mean value and the most extreme values in the range. An 82.5% confidence level means that the difference between the mean and the extreme values is 17.5%. Data were returned for 226 countries and territories. In related areas, this methodology was also used to track the emergence of the carbon market intelligence sectors (*15*) and spending in megacities on climate change adaptation (*14*), to estimate private sector research and development spending on clean energy (*32*), and by the UK Department for Business, Innovation and Skills for reporting on the low carbon and environmental goods and services sector (*27*).

### Country and regional comparisons

To compare countries on development status, two classifications were used. The UNDP’s HDI assesses countries across a number of social and economic indicators (including life expectancy, mean years of schooling, and gross national income at purchasing power parity per capita) to arrive at a classification of development status with four tiers: very high, high, medium, and low (*33*). It is a classification that has been consistently developed to cover all countries and to take a wider view of development progress than assessments solely based on GDP. For comparison, the World Bank’s income classification (for the 2015/2016 fiscal year) was also used, which has five tiers: low, lower-middle, upper-middle, high non-OECD, and high OECD (*34*). It provides a useful comparison to the HDI because it has five levels of classification and it splits high-income countries between OECD and non-OECD. Used in concert, they can show evidence of differing trends based on their different stratifications of countries. GDP data were taken from the April 2016 update of the International Monetary Fund World Economic Outlook (*35*). The WCIS as a percentage of global GDP comparison was based on 185 countries and territories that returned both transactional spend on WCIS and International Monetary Fund World Economic Outlook 2015 GDP (nominal) data.

For regional comparisons, country classification by region was assessed according to the World Bank’s regional classification (*34*). This was preferred to other regional classifications, particularly to the UN Geoscheme (*36*), because it splits the world into seven regions with areas and populations within a rough order of magnitude: East Asia and the Pacific, Europe and Central Asia, Latin America and the Caribbean, Middle East and North Africa, North America, South Asia, and sub-Saharan Africa. The UN Geoscheme has five regions (Africa, Americas, Asia, Europe, and Oceania), which are too imprecise, and 22 subregions, which provides too many regions for consistent and clear analysis.

### Climate risk comparisons

To enable the country comparisons on climate risk, the Germanwatch Climate Risk Index 2016 was used as a measure of climate and extreme weather–related risks (*18*). The data collection was conducted by Munich RE’s NatCatSERVICE. Data collection covers “all elementary loss events which have caused substantial damage to property or persons” (*18*). On country-by-country basis, the NatCatSERVICE collects data on the number of total losses caused by weather-related events, number of deaths, insured damages, and total economic damages. This index provides a measure of exposure and vulnerability to climate-related risks over the period of 1995–2014 through the following indicators: death toll, death toll per 100,000 inhabitants, total losses in million dollars, and losses per unit GDP in percent. The indexing is weighted toward the relative indicators, death toll per 100,000 inhabitants and losses per unit GDP in percent, because this provides a more comparable measure of exposure and vulnerability. The index reflects direct impacts only rather than indirect impacts (such as droughts and food scarcity caused by heat waves).

### Data availability

There are variations in the availability of data and the definitions of countries, regions, and territories between different data sets used in this study. Therefore, the analysis presented in each figure is a broad subset of the total number of countries and territories, excluding countries from figures presented where no data are available. The number of countries and territories in each data source is as follows: kMatrix WCIS data (this study), 226 countries and territories; HDI, 188 countries; Climate Risk Index, 187 countries; World Bank’s income classification, 198 countries; World Bank’s regional classification, 198 countries; International Monetary Fund World Economic Outlook, 192 countries. A breakdown of the number of countries covered by each figure is provided in the figure legends.

## Supplementary Material

http://advances.sciencemag.org/cgi/content/full/3/5/e1602632/DC1

## SUPPLEMENTARY MATERIALS

Supplementary material for this article is available at http://advances.sciencemag.org/cgi/content/full/3/5/e1602632/DC1 fig. S1. Global map of country-by-country per capita spending on WCIS. fig. S2. Global map of country-by-country spending on WCIS as percentage of GDP. table S1. Breakdown of WCIS by data platform, service type, and industry/economic sector. table S2. Examples of WCIS transactions and their allocation to weather services and climate services. table S3. Two examples of the data classification taxonomy for legal and financial and manufacturing industries.
