# Supplementary material for: Global disparity in the supply of commercial weather and climate information services
Source: Sci Adv. 2017 May 24;3(5):e1602632. doi: 10.1126/sciadv.1602632 (PMC5443644; doi:10.1126/sciadv.1602632)
Supplement: http://advances.sciencemag.org/cgi/content/full/3/5/e1602632/DC1 [file supp_3_5_e1602632__index.html]

Science Advances | Science Advances

## Supplementary Materials

**This PDF file includes:**

- fig. S1. Global map of country-by-country per capita spending on WCIS.
- fig. S2. Global map of country-by-country spending on WCIS as percentage of GDP.
- Legends for tables S1 to S3

Download PDF

**Other Supplementary Material for this manuscript includes the following:**

- table S1 (Microsoft Excel format). Breakdown of WCIS by data platform, service type, and industry/economic sector.
- table S2 (Microsoft Excel format). Examples of WCIS transactions and their allocation to weather services and climate services.
- table S3 (Microsoft Excel format). Two examples of the data classification taxonomy for legal and financial and manufacturing industries.

**Files in this Data Supplement:**

- Adobe PDF - 1602632\_SM.pdf
