# Supplementary material for: Global disparity in the supply of commercial weather and climate information services
Source: Sci Adv. 2017 May 24;3(5):e1602632. doi: 10.1126/sciadv.1602632 (PMC5443644; doi:10.1126/sciadv.1602632)
Supplement: http://advances.sciencemag.org/cgi/content/full/3/5/e1602632/DC1 [file 1602632_SM.pdf]

Science Advances

AAAS

advances.sciencemag.org/cgi/content/full/3/5/e1602632/DC1

# Supplementary Materials for

## Global disparity in the supply of commercial weather and climate information services

Lucien Georgeson, Mark Maslin, Martyn Poessinouw

Published 24 May 2017, *Sci. Adv.* **3**, e1602632 (2017)
DOI: 10.1126/sciadv.1602632

**The PDF file includes:**

- fig. S1. Global map of country-by-country per capita spending on WCIS.
- fig. S2. Global map of country-by-country spending on WCIS as percentage of GDP.
- Legends for tables S1 to S3

**Other Supplementary Material for this manuscript includes the following:**
(available at advances.sciencemag.org/cgi/content/full/3/5/e1602632/DC1)

- table S1 (Microsoft Excel format). Breakdown of WCIS by data platform, service type, and industry/economic sector.
- table S2 (Microsoft Excel format). Examples of WCIS transactions and their allocation to weather services and climate services.
- table S3 (Microsoft Excel format). Two examples of the data classification taxonomy for legal and financial and manufacturing industries.

Supplementary Figure 1

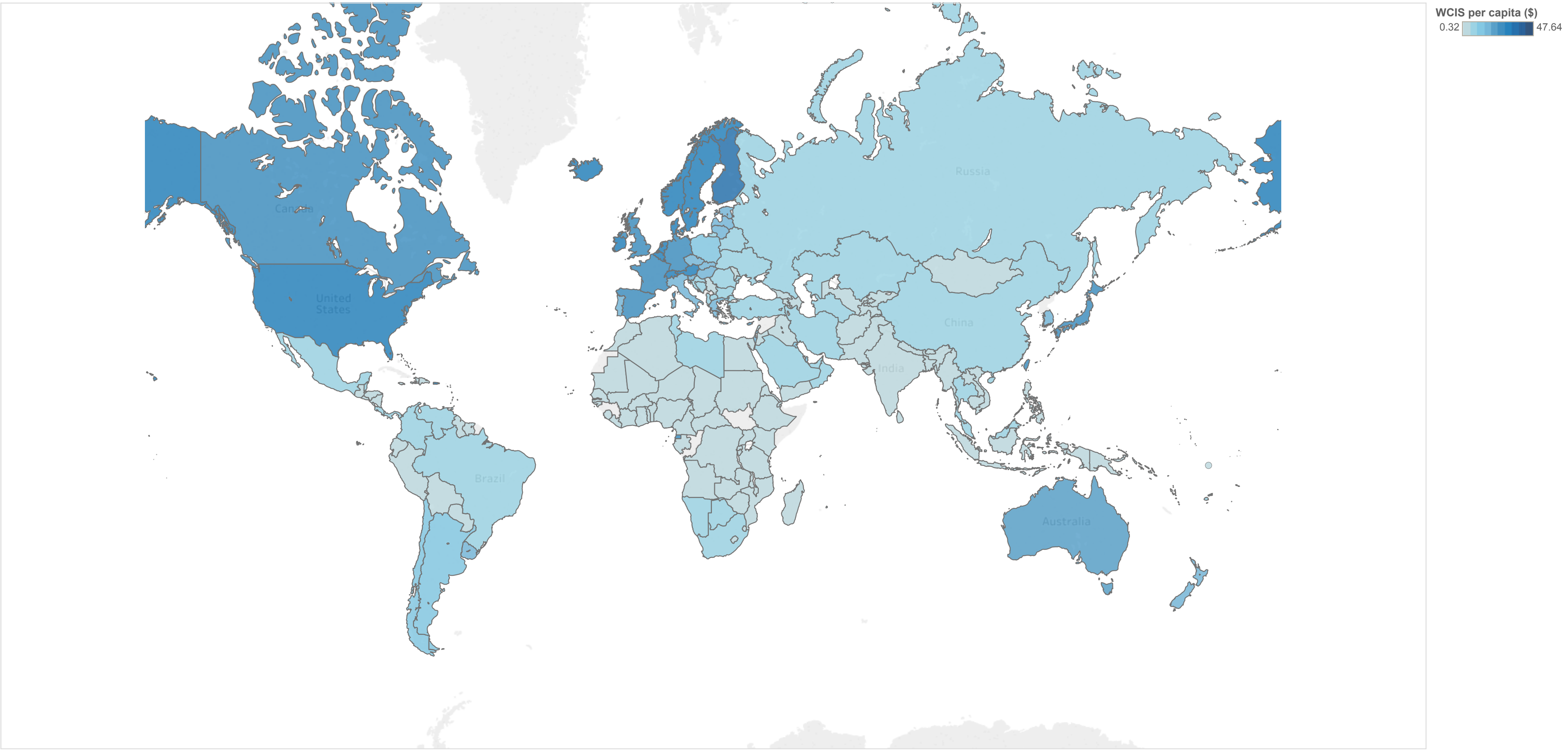


Map based on Longitude (generated) and Latitude (generated). Color shows sum of WCIS per capita ($). Details are shown for Country. The view is filtered on Latitude (generated), Longitude (generated) and sum of WCIS per capita ($). The Latitude (generated) filter keeps non-Null values only. The Longitude (generated) filter keeps non-Null values only. The sum of WCIS per capita ($) filter ranges from 0.00 to 47.64.

**fig. S1. Global map of country-by-country per capita spending on WCIS.**

Supplementary Figure 2

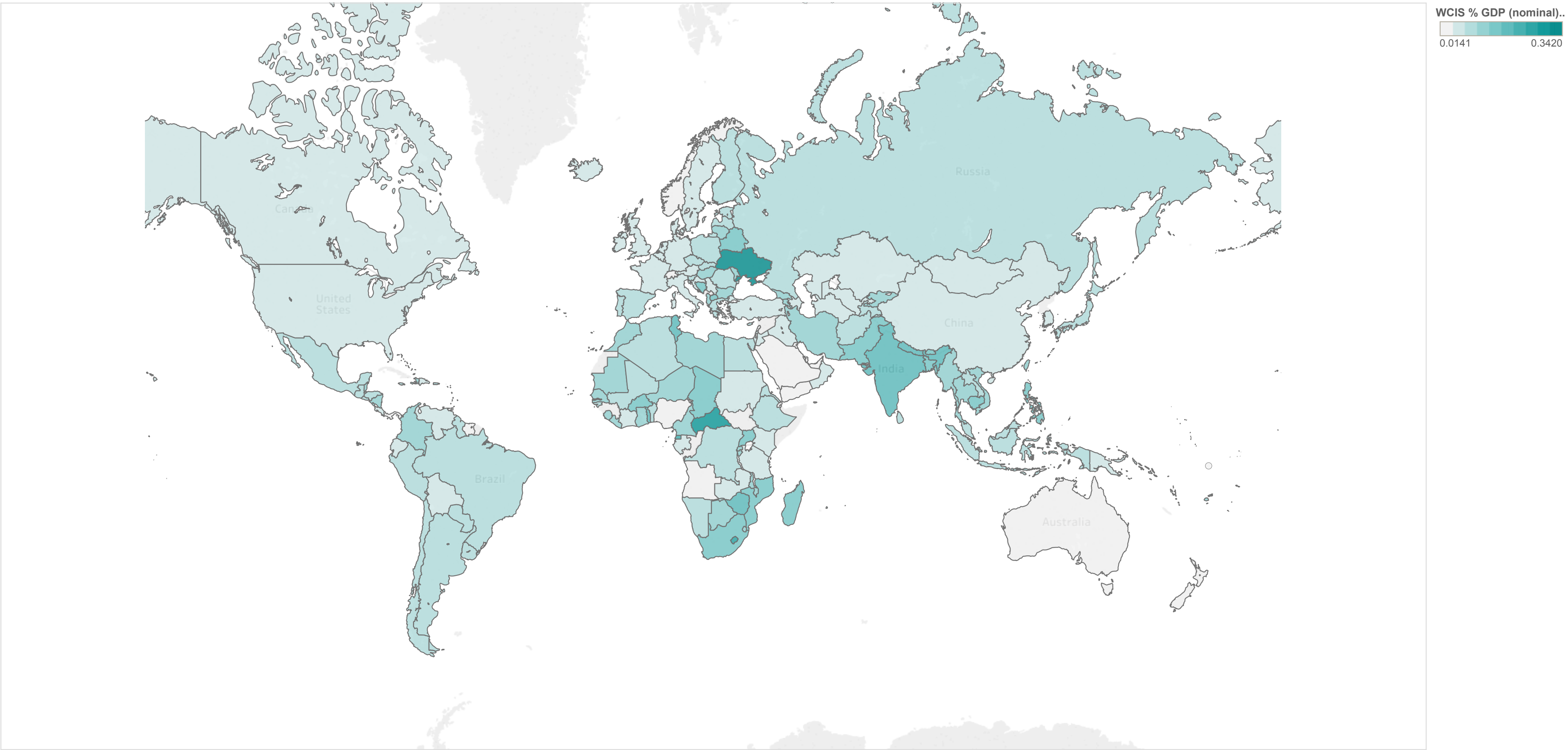


Map based on Longitude (generated) and Latitude (generated). Color shows sum of WCIS % GDP (nominal) ($). Details are shown for Country. The data is filtered on sum of WCIS per capita ($), which ranges from 1e-08 to 47.637699957. The view is filtered on Latitude (generated) and Longitude (generated). The Latitude (generated) filter keeps non-Null values only. The Longitude (generated) filter keeps non-Null values only.

**fig. S2. Global map of country-by-country spending on WCIS as percentage of GDP.**

**Supplementary Tables:**

**table S1. Breakdown of WCIS by data platform, service type, and industry/economic sector.**

**table S2. Examples of WCIS transactions and their allocation to weather services and climate services.**

**table S3. Two examples of the data classification taxonomy for legal and financial and manufacturing industries.** The structure of the data classification is the same across the four data platform categories (Space, Airborne, Land-based, and Marine In-Situ Services), and so one data platform example is given for each of the two industries.
